# Inflammasomes and Their Role in Innate Immunity of Sexually Transmitted Infections

**DOI:** 10.3389/fimmu.2016.00540

**Published:** 2016-12-05

**Authors:** Vivek Verma, Rakesh Singh Dhanda, Niels Frimodt Møller, Manisha Yadav

**Affiliations:** ^1^Dr. B. R. Ambedkar Center for Biomedical Research, University of Delhi, New Delhi, India; ^2^Department of Translational and Regenerative Medicine, Post Graduate Institute of Medical Education and Research (PGIMER), Chandigarh, India; ^3^Department of Clinical Microbiology, Rigshospitalet, Copenhagen, Denmark

**Keywords:** innate immunity, inflammasomes, NOD-like receptors, sexually transmitted infections, host defense, pathogens

## Abstract

Inflammasomes are multiprotein complexes present in the cytosol as pattern recognition receptors or as sensors of damage-associated molecular patterns. After recognition of microbe-associated molecular patterns or host-derived danger signals, nucleotide oligomerization domain-like receptors oligomerize to form inflammasomes. The activation of inflammasomes results in an alarm, which is raised to alert adjacent cells through the processing and release of a number of other substrates present in the cytosol. A wide array of inflammasomes and their adapter molecules have been identified in the host’s innate immune system in response to various pathogens. Components of specific pathogens activate different inflammasomes, which once activated in response to pathogen-induced infection, induce the activation of caspases, and the release of mature forms of interleukin-1β (IL-1β) and IL-18. Identifying the mechanisms underlying pathogen-induced inflammasome activation is important if we are to develop novel therapeutic strategies to target sexually transmitted infections (STIs) related pathogens. This information is currently lacking in literature. In this review, we have discussed the role of various inflammasomes in sensing different STIs, as well as the beneficial or detrimental effects of inflammasome signaling in host resistance. Additionally, we have discussed both canonical and non-canonical processing of IL-1β induced with respect to particular infections. Overall, these findings transform our understanding of both the basic biology and clinical relevance of inflammasomes.

## Introduction

Innate immune receptors act as the first line of defense against infectious microbes, continuously monitoring the extracellular milieu as well as subcellular compartments. These receptors can either be extracellular, such as some of the toll-like receptors (TLR) and C-type lectin receptors (CLR), or intracellular, such as nucleotide oligomerization domain (NOD)-like receptors (NLR), retinoic-acid-inducible gene (RIG-I)-like receptors (RLR), and AIM2 (absent in melanoma 2)-like receptors (ALR). Cytosolic immune receptors not only act as pattern recognition receptors (PRRs) that recognize pathogen-associated molecular patterns (PAMPs) but also sense signals derived from the host commonly known as damage-associated molecular patterns (DAMPs). These include intracellular molecules such as ATP and high-mobility group box 1 (HMGB1) protein as well as proteins derived from extracellular matrix. These PRRs trigger a downstream signaling cascade in the presence of specific ligands, resulting in the activation of transcription machinery inducing the production and release of pro-inflammatory cytokines. These cytokines further regulate the switch between tissue homeostasis and the inflammatory state, aimed at the removal of pathogens thus restoring normal tissue function (1).

Inflammasomes are multiprotein complexes consisting mainly of NLR, ASC (apoptosis-associated speck-like protein), and caspase-1, which are formed upon activation by specific ligands. NLRs are cytosolic protein receptors, and under highly regulated conditions, they assemble with ASC and caspase-1 to form speck-like aggregates (2, 3). The composition of inflammasomes varies in response to different ligands, for example, NLRP1 is a sensor of muramyl dipeptide (MDP: toxin of *Bacillus anthracis*) (4), NLRP3 responds to various cytosolic insults such as reactive oxygen species (ROS) (5), cathepsin B release (6), pore forming toxins [PFTs: nigericin (7)], extracellular crystals (8), and NLRC4 that oligomerizes in response to the presence of flagellin and PrgJ rod proteins within the cytoplasm (9). PYHIN (pyrin containing HIN200 domain) containing AIM2 and IFI16 (interferon-γ-inducible protein 16) initiates the formation of the inflammasome in response to foreign cytoplasmic double-stranded DNA (10, 11). Initially suggested as sensors of a microbial infection (12), inflammasomes have recently emerged as central orchestrators of microbial infections (13).

Aberrantly high inflammasome activation and abrogation of inflammasome signaling in different tissues leads to the development of various inflammatory pathologies, indicating the necessity of a finely balanced signaling cascade and a downstream cytokine release. Initial regulation of inflammasome activity occurs through transcriptional/posttranslational control. As a consequence of this, inflammasome expression levels are usually low in most cell types at the steady state. A stimulus (for example, extracellular PRR signaling) is required for induction of the inflammasome gene transcription and is known as signal 1 or priming (14, 15). Inflammasome formation then depends on the activation of specific NLR triggers, referred to as signal 2. Many potential inflammasome stimulators are now known, such as microbe-derived substances, molecules related to endogenous damage, and environmental particles (16).

Inflammasomes recruit and activate pro-inflammatory caspases, which result in the cleavage of pro-forms of IL-1β and IL-18 into mature and secretable forms. Each inflammasome contains ASC, an adaptor protein for caspase activation and a recruitment domain (CARD); however, NLRC4 contains a CARD domain that itself interact with caspase-1. Furthermore, inflammasome activity is tightly regulated since it leads to the production of some of the most potent pro-inflammatory cytokines (IL-1β and IL-18) and the subsequent induction of inflammatory cell death, pyroptosis (12). Inflammasome-mediated processes act as a key factor for clearance of infections and regulation of various metabolic and immune processes. In the past decade, inflammasome research has intensified with regards to various metabolic and infectious diseases. However, the mechanisms behind the stimulus of inflammasome assembly during sexually transmitted infections (STIs) are poorly understood.

Sexually transmitted infections are one of the most highly prevalent infections, with 340 million new cases every year worldwide, of which 50% are among adolescents and young adults (17). Most of these STI cases are reported in developing nations, mainly South Asia, Southeast Asia, Sub-Saharan Africa, Latin America, and the Caribbean (18). STIs impose huge economic losses every year because of its high incidence (19). STDs cost the U.S. health-care system an amount of $15.9 billion annually (20). Though STIs caused by bacterial, mycological, and protozoan agents can be treated successfully, all the viral STIs are not treatable with current medications. STIs not only cause intense morbidity in adults but also lead to intense complications such as infertility, ectopic pregnancy, fetal wastage, premature birth, low birth weight, premature mortality, cervical tumor, inborn syphilis, and ophthalmia neonatorum (21). The effect of inflammasome signaling during the development of various STIs has not been adequately studied. Here, we review the recent progress of our understanding of inflammasome signaling and the consequences of defective inflammasome activation during various STIs.

## *Chlamydia* Infection

*Chlamydia trachomatis*, an obligate intracellular Gram-negative human pathogen, is a leading cause of bacterial STI and preventable blindness worldwide. *C. trachomatis* infects both men and women and inflicts a wide range of diseases including conjunctivitis, urethritis, ectopic pregnancy, and infertility in the affected women. Infection of pregnant women can be passed on to the baby during pregnancy or childbirth, causing conjunctivitis or fatal pneumonia in the newborn. *Chlamydia* affects 90 million new cases each year worldwide (22).

The pathogenesis of the *Chlamydia* infection and the resulting inflammatory process have been examined using multiple animal models and *in vitro* studies (22). The infection is initiated by the attachment and entry of elementary bodies (EBs) into epithelial cells that transit into replicative reticulate bodies (RBs) to establish a parasitophorous vacuole, also known as inclusion (23). *C. trachomatis* employs a type III secretion system (T3SS) and encodes a chlamydial protease-like activity factor (CPAF) to establish a replicative niche within host cells (24, 25). Response to an epithelial cell infection occurs within a few days and is characterized by neutrophil infiltration, which kills accessible EBs, followed by an accumulation of T-cells and other leukocytes in the infected area (26). The inflammatory process resulting from primary infection often results in either long-term tissue damage or tubal damage and infertility (22).

*Chlamydia trachomatis* activates the NLRP3 inflammasome in an ASC and caspase-1-dependent manner in diverse human and mouse cells (27–30). However, the role of non-NLRP3 inflammasomes during infection cannot be totally refuted. In a recent animal study, a murine model of *Chlamydia muridarum* infection shows interferon (IFN)-inducible guanylate-binding protein (GBP)-dependent pyroptosis through activation of caspase-11-dependent non-cannonical and caspase-1-dependent cannonical inflammasomes (NLRP3 and AIM2) (31). In *NLRP3*^−/−^- and *ASC*^−/−^-deficient mice, survival of *Chlamydia* and caspase-1-dependent IL-18 secretion (32) were reduced, and lipid droplets were accumulated showing evidence of promotion of atherosclerosis and metabolic diseases (33). In another study, *ApoE*^−/−^ (Apolipoprotein E)-deficient mice fed on high fat diet and infected with *Chlamydia* resulted in accelerated atherosclerosis with a presence of activated myeloid DCs (mDCs) and plasmacytoid DCs (pDCs) (34). In a report from Nagarajan et al., mice deficient in IL-1R showed delayed clearance of *Chlamydia*, supporting the role of IL-1β in infection clearance, but they suggested a low significance of inflammasome pathways in IL-1β secretion and genital tract pathologies (32). Particularly pORF5, a secretory protein of *Chlamydia* sp. has been found to facilitate the secretion of IL-1β and IL-18 through NALP3 activation (35).

In mouse bone marrow macrophages, the AIM2 inflammasome contributes partially to the production of IL-1β and IL-18 during the infection (31). Thus, prior exposure of *C. trachomatis*-infected THP-1 cells to anti-oxidants partially inhibits IL-1β secretion, reinforcing the fact that multiple upstream mechanisms or inflammasomes may be involved (30). Thus, multiple pathways including non-TLRs contribute to IL-1β production equally. Evidence suggests a role of NOD1 in IL-1β secretion from a human trophoblast cell line exposed to *Chlamydia* (36).

**Figure 1:**
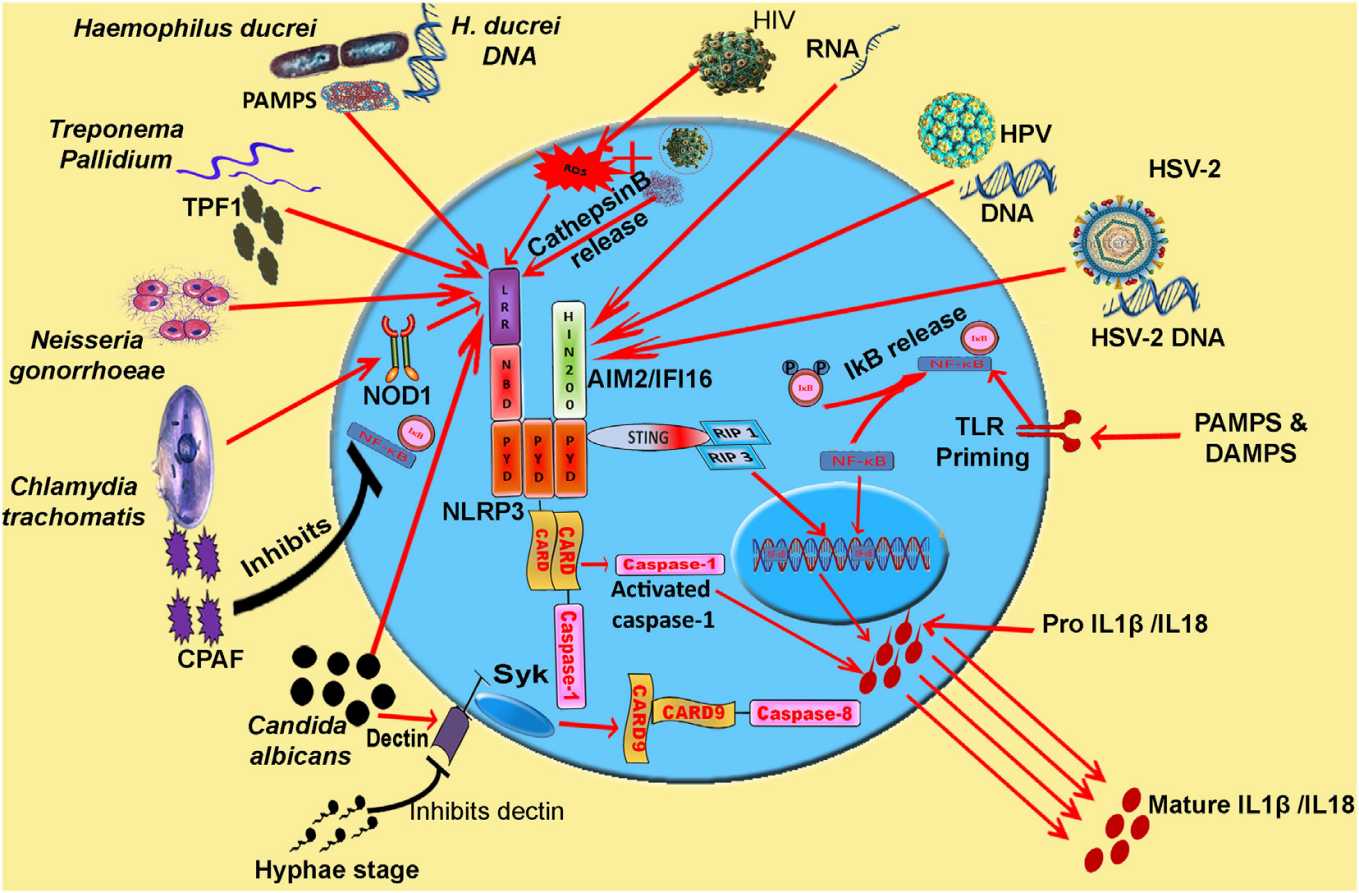
**Model: inflammasome activation and pro-inflammatory cytokine release during various STIs**. Synchronization of various inflammasomes and their adapters in the regulation of pro-inflammatory cytokine release. Cytosolic DNA and viral RNA of various viruses (HIV, HSV-2, and HPV) and different pathogens are sensed by AIM2 inflammasome. PAMPs and DAMPs of *Haemophilus ducreyi, Neisseria gonorrhoeae*, and TPF1 (*Treponema pallidum* factor-1) of *Treponema pallidum* trigger NLRP3 inflammasome. CPAF protease of *Chlamydia trachomatis* inhibits NF-κB signaling and pathogen triggers NLRP3 signaling *via* Nod 1 adapter. IL-1β release resulting from *Candida albicans* infection is mediated by caspase-8 activation. Abbreviations: NLRP, Nod-like receptor protein; NLRC4, Nod-like receptor containing CARD4; AIM2, absent in melanoma 2; IFI16, interferon inducible; LPS, lipopolysaccharide; MDP, muramyl dipeptide; PFTs, pore forming toxins; ROS, reactive oxygen species.

Contrary to the protective function of the NLRP3 upon infection with the respiratory pathogen *Chlamydia pneumoniae* (37), caspase-1 activation promotes the growth of *C. trachomatis* in cervical epithelial cells (29). The finding mentioned in the former study is supported at least partially by the observed inflammasome-mediated pyroptosis of *Chlamydia*-exposed antigen presentation cells, which are critical in *Chlamydia* clearance (38). However, a later study has its own limitations as the results were obtained in *IL-10*^−/−^ dendritic cells (DCs) and no direct observations were made in cells lacking inflammasome components. Nevertheless, *IL-10* deficiency does result in enhanced inflammasome activation (39, 40). Contrary to the results in cervical epithelial cells, *Caspase-1*^−/−^ mice exhibit similar *C. trachomatis* growth in the urogenital tracts (41). However, a *Caspase-1* deficiency results in significantly reduced inflammatory damage, suggesting that inflammasomes may not be directly controlling the *C. trachomatis* infection *in vivo per se*, but caspase-1 activation can invoke the *Chlamydia*-induced inflammatory pathologies in the genital tract (41) (Table 1; Figure 1).

**Table 1:** **Different inflammasomes activated during STIs and their respective stimulus**.

S. no.	Organism/disease	Inflammasome activated	Ligands and activation stimulus	Experimental model	Reference
1	*Chlamydia trachomatis*	NLRP3, NOD 1	Type third secretion system (T3SS), chlamydial protease-likeactivity factor (CPAF)	Monocytes, dendritic cells	(27–30)
2	*Candida albicans*: candidiasis	NLRP3, NLRC4	Hyphae formation induces IL-1β secretion, syk signaling activates NLRP3 through CARD9, *C. albicans* infection activated NLRC4 in mucosal stromal cells	Monocytes-derived macrophages and dendritic cells	(42–44)
3	HIV: AIDS	NLRP3	Pro IL-1β expression occurs through TLR8-dependent mechanism and IL-1β processing is done by NLRP3 activation, which occurred by HIV-1-induced ROS generation and cathepsin B release from disrupted lysosomes	Thp1-derived macrophages and peripheral blood mononuclear cells	(45)
4	Herpes simplex virus: genital herpes	IFI16 (AIM2-like receptors)	Cytosolic DNA sensed by IFI16	Thp1-derived macrophages	(11)
5	Human papilloma virus	IFI16 and AIM2, NLRP3	IFI16 and AIM2 sense viral DNA present in cytosol, Cathepsin B release and ROS generation activates NLRP3	Keratinocytes, Thp1 cells	(46, 47)
6	*Haemophilus ducreyi*: chancroid	AIM2 and NLRP3	ROS generation, K^+^ efflux, Cathepsin B activity, and lysosome acidification	MDM, Thp1-cells	(48)
7	*Neisseria gonorrhoea*: gonorrhea	NLRP3	*N. gonorrhoea* activates Cathepsin B which is responsible for NLRP3	Thp1, PBMC-derived macrophages	(49)
8	*Treponema pallidum*: syphilis	–	Activation by extracellular ATP followed by binding to P2Ξ7 receptor	PBMC-derived macrophages	(50)

### Candidiasis

Candidiasis is a well-known fungal infection caused by the yeast *Candida* with more than 20 different types known so far. *Candida albicans* is a well-tolerated resident of mucosal surfaces of the gastrointestinal and urogenital tract in most healthy people. However, an overgrowth of *C. albicans* can lead to an invasion of the epithelium causing oropharyngeal candidiasis (thrush) or genital/vulvovaginal candidiasis (yeast infection). The “yeast infection” is so common that nearly 75% of all adult women have at least one infection in their lifetime. Additionally, during primary and/or secondary immunodeficiency, yeast is the primary cause of opportunistic infections causing mucocutaneous candidiasis and occasionally systemic sepsis (51).

The cell wall architecture of *Candida* is well characterized and is deemed essential for the biology and pathogenicity of *C. albicans* (52). It consists of three basic constituents: β-glucans, chitin, and mannoproteins. β-glucans and chitin represent the structural components and provide physical properties to the cell wall (52). β-glucans and mannans display the most potent immunomodulatory activity leading to NLRP3 inflammasome activation and IL-1β secretion in mouse macrophages and human monocytes (53). The transcriptional upregulation of IL-1β is attained by Dectin-1, TLR2, and mannose receptor signaling upon recognition of specific *Candida* cell wall components, known as signal 1 in case of candidiasis (54). Inflammasome-mediated IL-1β secretion is essential in mounting a protective Th17 response that is central for the activation of mucosal antifungal immunity (55–57). In agreement, mice inept at sensing *C. albicans* demonstrate an enhanced dissemination of a mucosal infection and significant mortality *in vivo* (58). Activation of the NLRP3 inflammasome is mediated by a two-signal mechanism. However, the tyrosine kinase syk, functioning downstream of Dectin-1, couples both the two signals during a *C. albicans* infection. The syk/CARD9 pathway upregulates IL-1β production (59, 60), while syk-mediated ROS production and potassium efflux enable caspase-1 and inflammasome activation, resulting in IL-1β maturation (53). These studies suggest that syk and NLRP3 are critical in controlling *C. albicans* infection. Mice lacking either syk or NLRP3 demonstrate an enhanced fungal burden and mortality (53, 61). Furthermore, targeted deletion studies suggest a key importance of syk in CD11c^+^ cells during systemic candidiasis (61) implying that NLRP3 activation in CD11c^+^ cells governs candidiasis.

Ligation of dectin-1 with specific antigens assembles inflammasomes independent of caspase-1. Exposure of DCs to curdlan and certain *C. albicans* strains assembles the Dectin-1 triggered CARD-9–Bcl-10–MALT1 scaffold for IL-1β transcription. At the same time, Dectin-1 initiates recruitment of MALT1–caspase-8 and ASC into this scaffold leading to caspase-8 (56)-dependent pro-IL-1β cleavage. This suggests that distinct downstream signaling pathways can possibly also be triggered through the same receptor suggesting the diversity of immune pathways and their activation mechanisms. It is likely that the affinity of receptor–ligand interactions dictates these discrete signaling outcomes. Contrary to the previous belief, the caspase-8/NLRP3 inflammasome can be initiated independent of pathogen internalization. However, activation of NLRP3 is ambiguous in this setting, and it is possible that either fungi or their secreted toxins progress to the cytoplasm. The emergences of non-canonical and non-redundant inflammasomes for pro-IL-1β processing emphasize the diversity and versatility of the immune response to pathogens (56).

The precursor form of IL-1β can be cleaved by several proteases; however, it is still surprising that CARD-containing caspase-8 can substitute for caspase-1 in the inflammasome considering that no other caspase family member is known to do so (62). Secreted aspartic proteases (Sap), a family of extracellular proteases released by *Candida*, also triggers IL-1β and IL-18 maturation dependent on K^+^ efflux and ROS production (44). Particularly, Sap 4–6 proteases activate NLRP3-triggered IL-1β secretion in vaginal fluid and recruit polymorphonuclear neutrophils (PMNs) during *C. albicans* intravaginal challenge (63). On the other hand, endocytosis of Sap stimulates the caspase-11 inflammasome through type I IFN signaling (64). Thus, Sap proteins serve as trigger factors for both canonical and non-canonical inflammasomes.

Dimorphic *Candida* switches from the yeast form (blastoconidia) to filamentous forms (hyphae) during decreased oxygen tension or limiting nutrients (65). Filamentous hyphal forms of *Candida* are considered virulent since they facilitate adhesion and penetration of epithelial and endothelial cells (65). The hyphal form has also been suggested to be essential for the activation of the NLRP3 inflammasome (42). However, whether this is sufficient enough is not clear since certain morphologically similar mutants induce lower levels of IL-1β than wild-type *C. albicans*. More importantly, certain mutants defective in hyphae formation trigger comparable levels of IL-1β secretion, thus implying that other microbial and host mechanisms are more likely to contribute to activating inflammasome assembly (66). In a murine model of oropharayngeal candidiasis, NLRP3 in either the hematopoietic/stromal compartments and epithelial NLRC4 play an important role in immune resistance to *Candida*, by mounting pro-inflammatory and antimicrobial responses (43). However, whether this is indeed true during intravaginal challenge is still not clear. It has been argued that recurrent episodes of symptomatic infection in women, which are associated with immune hyperreactivity to the fungus lead to exaggerated pathological inflammation and a robust vaginal PMN migration. This is driven by NLRP3 and epithelial NLRC4 activity, which is important during vaginal challenge and ineffective inflammation. A robust vaginal PMN migration occurs in susceptible women, promoting pathological inflammation without affecting fungal burden due to NLRP3 activation (67). Thus, during a vaginal challenge, both NLRP3 and NLRC4 were activated. While NLRP3 contributes to neutrophil recruitment and pathogenic inflammation, IL-22-induced pNLRC4 restricts NLRP3 by a sustained production of IL-1Ra (67). This suggests that while NLRP3 activity is critical in controlling systemic dissemination of the infection, NLRC4 activity in stromal cells is required for the protection from mucosal candidiasis (Table 1; Figure 1).

### Human Immunodeficiency Virus

Human immunodeficiency virus (HIV) is a retrovirus (ssRNA virus) that causes persistent inflammation and acute immune activation, which is a primary characteristic of this disease that contributes to the development of acquired immune deficiency syndrome (AIDS) (68, 69). The virus causes an infection with the transfer of body fluids from an infected person to another, which includes semen, vaginal fluids, breast milk, and blood. In its new host, the virus uses the envelope glycoprotein gp120 to attach to a CD4 receptor found on T-cells, monocytes, and macrophages. Around 35 million individuals are infected with HIV worldwide (70).

Polymorphisms in genes encoding NLRP3 and IL-1β have been observed in susceptible patients (71). However, the transcriptional profile of genes encoding inflammasome components in HIV patients is still under debate. HIV-positive patients display a similar transcriptional expression of inflammasome genes in peripheral blood mononuclear cells (PBMCs) compared with controls (72). However, *ex vivo* studies show that DCs obtained from HIV-1 patients are unresponsive as compared to an elevated mRNA expression of NLRP3, caspase-1, and IL-1β in control DCs (73). Having said that patient DCs possess an enhanced basal expression of NLRP3 and the unresponsiveness to a subsequent infection might be due to the saturation in NLRP3 gene levels or due to a chronic inflammatory state of these cells (73).

In monocytes and macrophages, HIV-1 is sensed by TLR8 leading to inflammasome activation, and this is not accompanied by type I IFN production (45, 74). This is an infection-independent mechanism and reveals utilization of distinct TLRs by different cell types for type I IFN or inflammasome pathways of inflammation. Interestingly, HIV-1 replication steps including entry, reverse transcription and integration, but not virion maturation, were all required, and HIV-1-induced ROS and cathepsin B also were necessary for the activation of NLRP3 inflammasome (45). The primary cause for progression to AIDS is the progressive loss of CD4 T cells, which might be due to inflammasome-induced pyroptosis, an inflammatory form of programed cell death (75). Cell-to-cell transmission of HIV is required for activation of caspase-1-dependent pyroptosis, and cell-free HIV-1 virions fail to induce pyroptosis (76) (Table 1; Figure 1).

### Genital Herpes

A crucial viral disease caused by herpes simplex virus type 1 (HSV-1) and type 2 (HSV-2) is known as genital herpes. Oral herpes is the most common form of this disease with visibly clear symptoms such as facial or oral infections and cold sores. Genital herpes is the second most common form of herpes infection (77). The specific clinical characteristics of herpes infections are painful ano-genital ulceration. HSV-1 and HSV-2 are the cause of oro-labial ulcers and ulcerative lesions, respectively, in adult populations (77). HSV-1-infected cells mediate the induction of IFN in which IFI16 and the murine protein p204 play an indispensable role (11). During HSV-2 infection, STING activates the NF-κB pathway (signal 1) to produce pro IL-1β through RIP1 and RIP3 (receptor-interacting protein). The viral DNA is sensed by the IFI16 inflammasome (signal 2), which oligomerizes to activate caspase-1 to convert pro-IL-1β into mature IL-1β (Table 1; Figure 1).

### Human Papilloma Viruses

The human papilloma viruses (HPVs) are non-enveloped, double-stranded DNA viruses that are the principle cause of benign warts and infections of the ano-genital region. Recent advancements in HPV-associated research led to the discovery of approximately 180 types of HPVs (78). The most common HPVs such as HPV-6 and HPV-11 constitute a 100% protective quadrivalent vaccine agent against genital warts infection (79).

A human papilloma virus activates the AIM2 inflammasome through viral DNA recognition. Additionally, another DNA sensor, IFI6, is activated resulting in IFN-γ release. The presence of the two DNA sensors and the active forms of caspase-1 and IL-1β are detectable in HPV-positive skin lesions, in which the virus selectively infects keratinocytes in stratified epithelia. However, the two DNA sensors seem to negate the effect of the other as blocking one of the sensors potentiates responses from the other in experiments performed in normal human keratinocytes (46). Whether and what role HPV does play in these processes is unclear from the current studies and a detailed understanding of these molecular pathways is required to develop improved strategies for the prevention and treatment of HPV infections (Table 1; Figure 1).

### Chancroid

*Haemophilus ducreyi* (Gram-negative, cocci) is a causative agent of the genital ulcer disease known as chancroid. The disease is prevalent in certain underdeveloped regions of Africa, Asia, and the Caribbean. Infection levels are very low in the developed countries and are mainly reported among individuals who have traveled outside these countries to affected regions.

*Haemophilus ducreyi* infection activates the inflammasome in monocyte-derived macrophages and experimentally infected human skin (48). Secretion of NLRP3-dependent IL-1β in macrophages is dependent on both caspase-1 and caspase-5. However, these results have been observed with caspase-1 and caspase-5 inhibitors, and further studies using genetic knockdown tools are required to substantiate this data. Interestingly, this activation is limited to M1 and M2 macrophages while non-polarized macrophages fail to activate inflammasomes, suggesting that the precise contribution of inflammasomes in human lesions cannot be directly extrapolated (48) (Table 1; Figure 1).

### Gonorrhea

*Neisseria gonorrhoeae* is a Gram-negative diplococcus that can grow and rapidly multiply in the mucous membranes especially in the mouth, throat, anus, cervix, fallopian tubes, and uterus of the infected individuals. Gonorrhea represents an estimated 60 million cases of urethritis and cervicitis every year around the world (80).

Even before inflammasome was first characterized, distinct immortalized epithelial cell lines from human endocervix, ectovervix, and vagina were demonstrated to secrete IL-1β and other pro-inflammatory cytokines when exposed to piliated *N. gonorrhoeae* (81, 82). Later studies revealed the identity of the NLRP3 inflammasome involved in causing pyronecrosis and HMGB1 release in macrophages in a cathepsin B-dependent manner. Hexa-acylated lipid A lipooligosaccharide, a *N. gonorrhoeae* virulence factor, activates the NLRP3 inflammasome (83). However, the phagosomal disruption and penetrance of host cytosol by *N. gonorrhoeae* as possibly activating NLRP3 cannot be completely ruled out (49) (Table 1; Figure 1).

### Syphilis

*Treponema pallidum*, a spirochete bacterium causing syphilis, is a well-known STI. Despite the fact that the essential course of transmission is through sexual activities, it is also transmitted to the fetus from mother either during pregnancy or at the time of childbirth, thus increasing chances of induced congenital syphilis. Protease resistant antigen *T. pallidum* factor-1 (AgTpF1) is found to be involved in the stimulation of monocytes resulting in the release of pro-inflammatory cytokines such as TNF-α, IL-6, and IL-1β. The IL-1β release was found to be particularly due to the inflammasome activation at early stages of syphilis infection (50). In a syphilis infection, Bacterioferritin protein TpF1 of *T. pallidum* activates the NLRP3 inflammasome to mediate the cleavage of pro IL-1β/18 into a mature and bioactive form of IL-1β/18 (Table 1; Figure 1).

### Urinary Tract Infection

Urinary tract infections (UTIs) are mostly caused by uropathogenic *Escherichia coli* (UPEC). UTIs affect at least 60% of all women once in their lifetime. UPEC virulence includes proteins that function in immune evasion, fimbriae, toxins, iron uptake, and biofilms. Innate immune responses are induced by neutrophils, antimicrobial peptides, and PRRs such as NLRs.

Despite several studies of the function of TLRs in UTI, the role of NLRs in UTI has not been elucidated. One of the few studies in the murine model of cyclophosphamide (CP) induced cystitis, glyburide suggested to suppress caspase-1 with reduction in the release of IL-1β; whereas NLRP3 inhibition blocks bladder dysfunction in CP model (84). The coordinated response of NLRs and TLRs in the urothelia represented a first line of innate defense. Another study identified a protective role of autophagy gene *ATG16L1* deficiency in UPEC-induced UTIs (85) and inflammasome activation is known to inhibit autophagy by regulating the secretion of IL-1β. NOD2 was found to be dispensable in the pathogenesis of UTIs in mice as well. Species and strain-specific inflammasome activation was found to be partially dependent on NLRP3 in human macrophages and completely in mouse macrophages (86).

## Conclusion and Future Perspectives

While our understanding of inflammasomes and their various regulatory mechanisms has increased tremendously in the last decade, we have only scratched the surface in terms of inflammasome activation during urinary and STIs. UTI-causing species belonging to genus *Enterococcus, Klebsiella*, and *Staphylococcus* induce NLRP3 inflammasome activation leading to excessive secretion of IL-1β and cell death in macrophages (87–91). However, the role of inflammasomes and the mechanism behind their activation are still unknown, due to the limited knowledge in this field. This is partly due to the lack of appropriate animal models available to study UTIs and STIs. However, many laboratories are now actively working on the function of NLR in innate immunity of the UTI patients. It is essential to understand which inflammasomes play a critical role in UTIs and STIs; what their activation mechanisms are; and how these multiprotein complexes sense a wide array of alterations in a cell type-specific manner. This knowledge will potentially lead to an improved therapeutic and diagnostic approach for treating a range of infections.

## Author Contributions

MY and VV: conceived and designed the idea. VV: online searches and data collection. MY, VV, RSD, and NFM: preparation of manuscript, and editing and proofreading of final manuscript.

## Conflict of Interest Statement

The authors declare that the research was conducted in the absence of any commercial or financial relationships that could be construed as a potential conflict of interest.
